# A New Computational Method for Detecting Leak Flow and Tidal Volume Waveforms During Spontaneous or Mandatory Breathing Assisted with Nasopharyngeal Ventilation

**DOI:** 10.3390/s25072022

**Published:** 2025-03-24

**Authors:** Francesco Montecchia, Paola Papoff

**Affiliations:** 1 Medical Engineering Laboratory, Department of Civil Engineering and Computer Science Engineering, University of Rome “Tor Vergata”, 1 Politecnico Street, 00133 Rome, Italy; 2 Pediatric Intensive Care Unit, Department of Pediatrics, Sapienza University of Rome, 00100 Rome, Italy; paola.papoff@uniroma1.it

**Keywords:** respiratory analysis, fluid dynamic model, computational modeling, processing algorithm, medical applications, medical devices

## Abstract

Nasopharyngeal ventilation (NPV) is a common technique used to support breathing, particularly when a patient’s respiration is inadequate, such as under sedation. It involves delivering oxygen through an endotracheal tube positioned above the glottis. Accurate tidal volume measurement is crucial for anesthesiologists, with the gold standard being a pneumotachograph. However, due to leakage from the mouth or mask, this method has limitations when applied to NPV. This study introduces a computational model that calculates respiratory flow in real time by accounting for leak flow. Results show that tidal volume measurements using this method are comparable to the gold standard, assuming the model’s assumptions hold true.

## 1. Introduction

In clinical practice, there are many situations that require ventilatory support by non-invasive ventilation (NIV), i.e., ventilation that does not demand endotracheal intubation of the patient. NIV can and should be used in all patients who are able to breathe spontaneously during most of the period of ventilatory support, but at a level that is insufficient to meet the physiologic demands [1,2].

NIV is delivered through various interfaces that connect the ventilator system to the patient’s airway. The most commonly used NIV interfaces in clinical practice are facial or nasal masks; nasal cannulae; endotracheal tubes inserted into the oropharyngeal cavity, i.e., nasopharyngeal tubes (NPTs); and helmets [2]. We refer to nasopharyngeal ventilation (NPV) when an NPT is used.

NPTs connected to a ventilator bag allow anesthesiologists to either assist spontaneous breathing or provide mandatory ventilation when the patient’s breathing is too shallow, as occurs under procedural sedation [3]. In addition, when combined with a specific algorithm, the NPT allows pharyngeal pressure calculation without the need to insert the additional cannula required for direct in situ pressure measurement. Such an algorithm, developed from a fluid dynamic model similar to the one described in the Materials and Methods (Section 2.3), has shown its feasibility in the field of pediatric anesthesia [4].

Unlike mechanical ventilation, where the leak flow at the interface occurs only when the cuff at the distal end of the endotracheal tube is not properly sealed to the inner wall of the trachea or is intentionally left deflated [5,6], during NIV, leak flow occurs systematically at all types of interfaces and cannot be avoided [1,2,7]. In addition, the magnitude of this leak flow cannot be predicted or estimated during treatment, so the output signal from the monitoring system’s sensors is not useful for accurate measurements of the respiratory flow waveform over time [8,9,10]. Specifically, because the flow sensor is placed upstream of the leak that occurs at the interface, its reading output overestimates or underestimates the instantaneous value of the true respiratory flow during inspiration and expiration, respectively. As a result, erroneous readings, if not corrected or compensated for, lead to unacceptable errors in the respiratory flow waveform over time and, consequently, in both the inspiratory and expiratory tidal volumes.

This error is not only a diagnostic problem, but also a therapeutic one, since the efficacy and safety of the NPV are strongly based on the correct determination of diagnostic parameters [11]. For these reasons, in all NIV modalities and for all types of interfaces and patients, it is essential to develop algorithms that allow the estimation of the leak flow waveform that occurs at the interface during breathing to be used to correct the measured respiratory flow waveform and ultimately to obtain the true respiratory flow waveform over time. Different types of algorithm have been proposed in this context, but none of them is compatible with NPT ventilation supported by the free-flow oxygen delivery device (FFODD) [12,13,14]. This is due to the impossibility of confining and therefore monitoring the patient’s upper airway from a fluid point of view, even partially, during clinical procedures associated with NPV. Among these algorithms, differential spirometry stands out, which is not feasible in NPV because it requires the use of a face mask equipped with a flow sensor [11,15,16,17].

The objective of this study is to delineate a novel computational methodology for respiratory flow monitoring in the clinical context of procedural sedation necessitating NPV, either in the spontaneous or mandatory mode. This methodology, facilitated by a designated algorithm, enables the calculation and real-time monitoring of the waveform over time of leak flow and, consequently, those of respiratory flow and tidal volume. The algorithm’s reliability was substantiated in an in vitro study that emulated the previously reported clinical scenario.

## 2. Materials and Methods

### 2.1. Description of the Ventilatory System (FFODD) Used with the NPT for NPV

**Figure 1:**
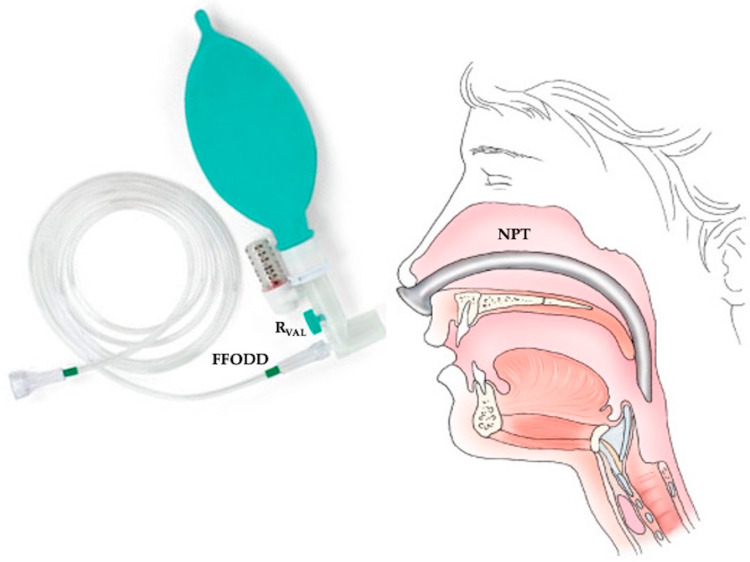
The FFODD is connected to the NPT, which is inserted through a nostril for NPV. The FFODD system has four pipes that are connected at the branch point. The first pipe is the inlet pipe. It receives the flow from the generator. The second pipe ends with a variable resistance valve (R_VAL_). The third pipe is the tube that fills the balloon. The fourth pipe connects to the NPT. The flow delivered by the generator is set based on the patient’s needs.

FFODD is used to apply either continuous positive airway pressure (CPAP) ventilation in the spontaneous mode through the NPT interface or mandatory ventilation [3]. Figure 1 shows a schematic representation of FFODD combined with NPT.

**Figure 2:**
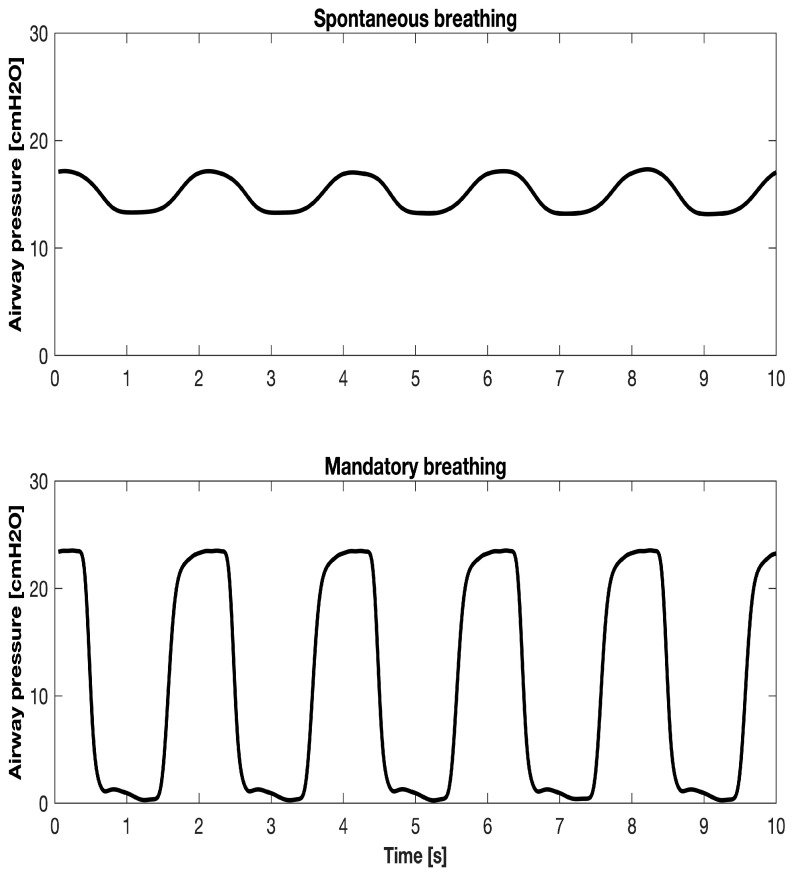
A typical airway pressure waveform over time during NPV. In the upper panel, the airway pressure waveform of spontaneous ventilation under CPAP is shown, whereas the lower panel shows mandatory ventilation.

The CPAP level depends on R_VAL_, flow rate, and leak flow. Spontaneous breathing causes the airway pressure to oscillate around the CPAP value, with inferior and superior pressures during inspiration and expiration. With mandatory delivery, the airway pressure increases above the CPAP level during inflation and decreases to the CPAP level during expiration. Figure 2 shows the typical airway pressure waveform over time, relative to spontaneous breathing and mandatory delivery.

### 2.2. Clinical Context and Experimental Setup Used to Measure and Calculate Leak Flow Waveform

The clinical context involves the simulation of a patient who has been sedated for a gastroscopy and is receiving NPV through an NPT. The patient’s breathing is simulated as either spontaneous or passive (Figure 2).

**Figure 3:**
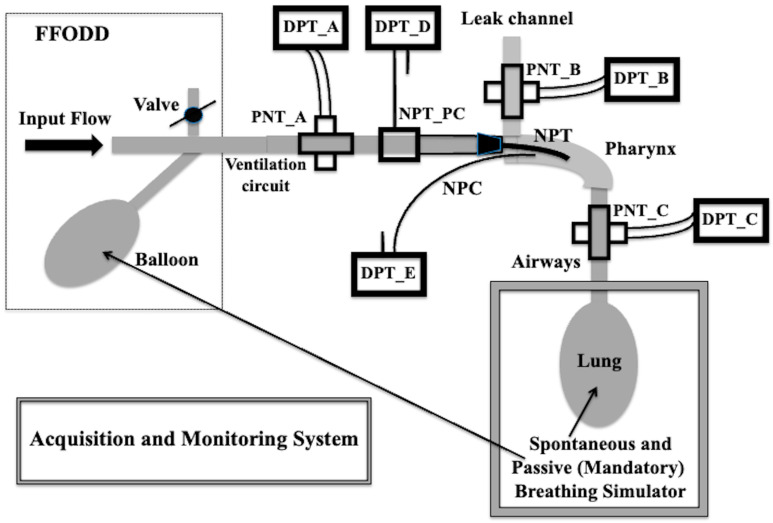
The experimental setup, including free-flow oxygen delivery device (FFODD), ventilation circuit, nasopharyngeal tube (NPT), nasopharyngeal pressure catheter (NPC), and leak channel, is used for the characterization of the relationship between pharyngeal pressure and leak flow and to calculate its fluidodynamic coefficients. When the lung simulator is included, the patient’s spontaneous or mandatory breathing is simulated so that leak flow, respiratory flow, and tidal volume can be calculated.

The corresponding experimental setup included the following units: FFODD, the NPT, the artificial pharynx, the leak channel from the pharynx, and the lung simulator (Figure 3). Using three pneumotacographs (PNTs) and five differential pressure transducers (DPTs), five measuring points monitored the quantities of interest (Figure 3). NPT flow (${\dot{V}}_{NPT}$) was measured by the PNT_A combined with the DPT_A, the leak flow (${\dot{V}}_{LEAK}$) by the PNT_B combined with the DPT_B, the NPT pressure (*P_NPT_*) by the DPT_D connected to the side aperture of the NPT pressure connector (NPT_PC), the respiratory flow (${\dot{V}}_{RES}$) by the PNT_C combined with the DPT_C, and the pharyngeal pressure (*P_PH_*) by the DPT_E. The DPT_A, DPT_B, and DPT_C are the SensorTechnics (SensorTechnics, Inc., Mansfield, CA, USA) 144LU01D-PCB transducers (full scale ±2.54 cmH_2_O), whereas DPT_D and DPT_E are the SensorTechnics (SensorTechnics, Inc., Mansfield, CA, USA) 144LU10D-PCB transducers (full scale ±25.4 cmH_2_O). The PNT_A, PNT_B, and PNT_C are the PNT series 3700 not heated (flow scale ±160 L/min), Hans Rudolph, Shawnee, KS, USA.

As NPTs, we used the 3.0, 4.0, and 5.0 mm internal diameter (ID) endotracheal tubes for children (Rusch, Teleflex Medical, Ireland) and the 7.0, 8.0, and 9.0 mm ID tubes for adults, all bent and kept folded to simulate the real curvature when inserted into the nasopharyngeal cavity. The Acquisition and Monitoring System used is the PicoScope 4824 equipped with its proprietary PicoScope 7 software (Pico Technology, Cambridgeshire, UK). The functional features of sensors and transducers and of the PicoScope 4824 are reported elsewhere [18]. An ASL 5000 Breathing Simulator (IngMar Medical, Pittsburgh, PA, USA) was used to simulate the spontaneous breathing or the ventilatory mandatory breaths. All the measurements were carried out using one of the most widely employed respiratory gas compositions in clinical practice with NPT ventilation, such as 100% oxygen and sevoflurane in oxygen at 2% concentration (2%Sevo/O_2_) (Sevorane, AbbVie Inc., North Chicago, IL, USA).

### 2.3. Physical Model of the Experimental Setup

**Figure 4:**
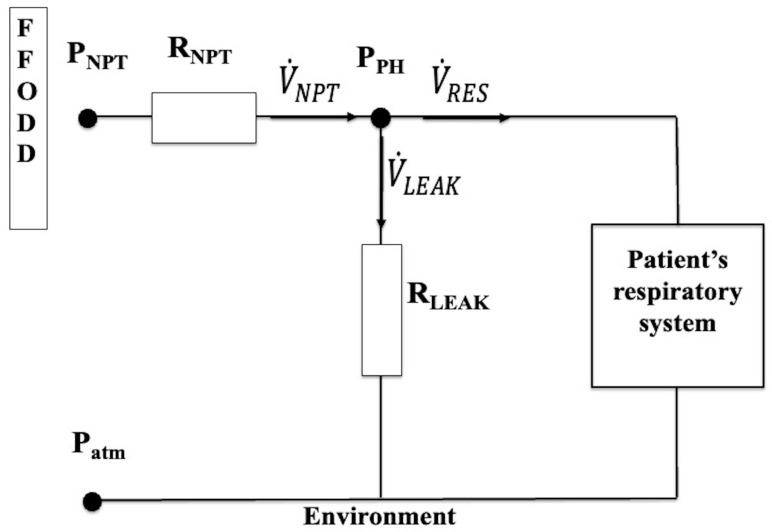
The equivalent electrical network of the physical model. The three pressure points (electric potential difference) relative to *P_NPT_*, *P_PH_*, and *P_atm_* are in bold. The three airflows (electric current) crossing the three branches (${\dot{V}}_{NPT}$, ${\dot{V}}_{LEAK}$, ${\dot{V}}_{RES}$) and the fluidodynamic resistance (electric resistance) of NPT (*R_NPT_*) and leak channel (*R_LEAK_*) are also shown.

We adopted the equivalence between fluidic and electric quantities. The equivalent electrical network of the physical model employed to represent the experimental setup (Figure 3) is reported in Figure 4.

Depending on the time point considered during spontaneous or mandatory breathing, the airflow (electric current) on all the branches of Figure 4 can be directed in both directions. The direction of the arrows shown in Figure 4 indicates the direction we arbitrarily chose as positive for the airflow on each branch: from the FFODD to the *P_PH_* node for ${\dot{V}}_{NPT}$, from the *P_PH_* node to the environment for ${\dot{V}}_{LEAK}$, and from *P_PH_* node to the patient’s respiratory system for ${\dot{V}}_{RES}$. In particular, with this setting, the inspiratory (into the patient) and expiratory (out of the patient) flows are positive and negative, respectively. Assuming the inflow into the node is positive and the outflow from the node is negative, applying the equivalent Kirchoff’s first or current law (also known as the continuity law) to the *P_PH_* node results in the following equation:

(1) $${\dot{V}}_{NPT}={\dot{V}}_{LEAK}+{\dot{V}}_{RES}$$

The pressure difference (ΔP) that occurs across two sections of any pipe when a flow ($\dot{V}$) of a real fluid passes through it generally consists of the following four components:

(1) Cinetic or Bernoulli component (ΔP_K_), which would exist if the fluid were ideal, since it only depends on the geometry;
(2) Viscous or resistive component (Δ*P_R_*);
(3) Elastic or capacitive component (ΔP_C_);
(4) Inertial component (ΔP_I_).

In NPV, a steady, adjustable flow is set along the NPT, which oscillates around the equilibrium value when the patient breathes spontaneously or when he/she is passively ventilated during apnea. The frequency of the oscillations (respiratory frequency) is always below 1–2 Hz (corresponding to 60–120 respiratory cycles per minute) and its amplitude is almost always lower than the set steady flow, so that the gas crossing the NPT almost never reverses its flow direction and almost never becomes negative, also due to the presence of a constant flow leak downstream of the NPT. For these reasons ΔP_I_ can be neglected. Moreover, the maximum value of *P_NPT_*, and therefore of *P_PH_*, is limited, so that even ΔP_C_ can be neglected, since the fluid can be considered incompressible (constant density), and the walls of the pipe are perfectly rigid. Finally, considering that the geometry of both the pipes involved (NPT and leak channel) and in particular the cross section sizes of the point where *P_NPT_* (connector at the proximal end of NPT, NPT_PC) is measured do not change during the assisted spontaneous breathing, ΔP_K_, which is not dependent on the value and type of flow (laminar, intermediate, or turbulent), can also be considered negligible because it does not affect neither the measurement of *P_PH_* by an NPC nor its calculation.

For all the aforementioned reasons, Δ*P_R_* was the only component of ΔP we considered across both *R_NPT_* and *R_LEAK_*_,_ the airflow fluidodynamic resistances appearing in Figure 4. Assuming that both laminar and turbolent regimes are present and that the ratio between ΔP_R_ and $\dot{V}$ is equal to the fluid dynamics viscous resistance (*R*) ($R=\frac{\Delta P_{R}}{\dot{V}}$), then *R* equals the sum of its laminar and turbolent components, which are non-dependent and linearly dependent on the $\dot{V}$, as it results from the following expression:

(2) $$R=k_{L}+k_{T}\;\dot{V}$$

where *k_L_* and *k_T_* are the laminar and turbolent coefficients related to R.

Considering Equation (2), *R_NPT_* and *R_LEAK_* assume the following expressions:

(3) $$R_{NPT}=k_{L\_NPT}+k_{T\_NPT}\;{\dot{V}}_{NPT}$$

(4) $$R_{LEAK}=k_{L\_LEAK}+k_{T\_LEAK}\;{\dot{V}}_{LEAK}$$

where *k_L_NPT_* and *k_T_NPT_* are the laminar and turbolent coefficients related to *R_NPT_*, while *k_L_LEAK_* and *k_T_LEAK_* are the corresponding coefficients related to *R_LEAK_*.

The application of the equivalent Kirchoff’s second or voltage law across *R_NPT_* and *R_LEAK_* yields the following equations:

(5) $$P_{NPT}=P_{PH}+(R_{NPT}\;{\dot{V}}_{NPT})$$

(6) $$P_{PH}=P_{atm}+(R_{LEAK}\;{\dot{V}}_{LEAK})$$

As *P_atm_* is assumed to be zero, Equation (6) yields the following equation:

(7) $$P_{PH}=R_{LEAK}\;{\dot{V}}_{LEAK}$$

Substituting *R_LEAK_* from Equation (4) into Equation (7) gives *P_PH_* as follows:

(8) $$P_{PH}=k_{L\_LEAK}{\dot{V}}_{LEAK}+k_{T\_LEAK}\;{\dot{V}}_{LEAK}^{2}$$

### 2.4. ${\dot{V}}_{LEAK}$ Calculation During Spontaneous or Mandatory Breathing

In order to calculate ${\dot{V}}_{LEAK}$ (see Equation (8)), it is first necessary to measure *P_PH_*. This can be accomplished by placing a pressure catheter within the pharynx (NPC, Figure 3). Once the *P_PH_* value has been established, the coefficients *k_L_LEAK_* and *k_T_LEAK_* must be calculated in order to extrapolate ${\dot{V}}_{LEAK}$.

#### 2.4.1. Method for *k_L_LEAK_* and *k_T_LEAK_* Calculation

**Figure 5:**
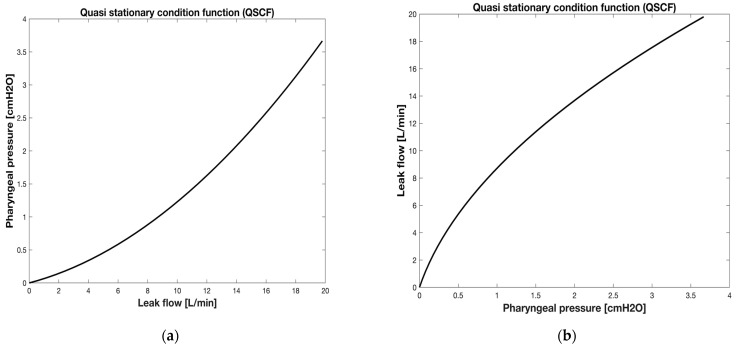
Rohrer’s equation (see Equation (8)) for the leak channel characterization *P_PH_* vs. ${\dot{V}}_{LEAK}$ (**a**) and ${\dot{V}}_{LEAK}$ vs. *P_PH_* (**b**). It is important to note that switching ${\dot{V}}_{LEAK}$ and *P_PH_* in the Cartesian axes (**b**) is useful in the calculation of the ${\dot{V}}_{LEAK}$ from the *P_PH_* (see Equation (10)).

The coefficients *k_L_LEAK_* and *k_T_LEAK_* are derived from Equation (8), which contains three unknown parameters: the coefficients *k_L_LEAK_* and *k_T_LEAK_* and the variable ${\dot{V}}_{LEAK}$. To overcome this challenge, a solution has been formulated as follows: if the patient is apneic, ${\dot{V}}_{LEAK}$ coincides at any time with ${\dot{V}}_{NPT}$, which can be measured by PNT_A (Figure 3). This condition can be achieved in vivo because the NPT is inserted after a small amount of sedative has been administered, causing the patient to become sedated and transitorily apneic, so that the gas flowing along the NPT (${\dot{V}}_{NPT}$) exits the pharynx and coincides with the leak flow (${\dot{V}}_{LEAK}$). Since ${\dot{V}}_{LEAK}$ during apnea is known, the two coefficients *k_L_LEAK_* and *k_T_LEAK_* can be easily calculated. More specifically, the method starts with building the relationship between the flow rate across the leak channel (${\dot{V}}_{LEAK}$)—increased from zero to 20 L/min (0.5 L/min increments) for children and from zero to 50 L/min (0.5 L/min increments) for adults—and the relative increase in *P_PH_*. This process is conducted in a stepwise manner. Both *P_PH_* and ${\dot{V}}_{LEAK}$ data are plotted one against the other (Figure 5a), yielding a set of points which are interpolated by means of the Rohrer equation ($∆P=k_{1}{\dot{V}}^{2}+k_{2}\dot{V}$) [19] applied to the leak channel (Equation (8)). The following step consists in substituting ΔP in the Rohrer equation with *P_PH_*, and $\dot{V}$ with ${\dot{V}}_{LEAK}$, yielding Equation (8). The interpolation with a second-order polynomial allows for the determination of both the coefficients *k_L_LEAK_* and *k_T_LEAK_* appearing in Equation (8).

During apnea, the condition that characterizes the ventilatory system can be defined as quasi stationary (QSC), as can the relationships *P_PH_* vs. ${\dot{V}}_{LEAK}$ (Figure 5a) and ${\dot{V}}_{LEAK}$ vs. *P_PH_* (Figure 5b), which is the quasi stationary condition function (QSCF).

#### 2.4.2. ${\dot{V}}_{LEAK}$ Calculation During Spontaneous or Mandatory Breathing Once *k_L_LEAK_* and *k_T_LEAK_* Coefficients Have Been Calculated During the Apnea

Once *k_L_LEAK_* and *k_T_LEAK_* have been calculated from the relationship between *P_PH_* and ${\dot{V}}_{LEAK}$ at the apnea time, *P_PH_* can be measured at any time during breathing and ${\dot{V}}_{LEAK}$ can be calculated accordingly using Equation (8), duly rewritten:

(9) $$k_{T\_LEAK}\;{\dot{V}}_{LEAK}^{2}+k_{L\_LEAK}{\dot{V}}_{LEAK}-P_{PH}=0$$

${\dot{V}}_{LEAK}$ can be extrapolated from the solution of Equation (9):

(10) $${\dot{V}}_{LEAK}=\frac{-k_{L_{LEAK}}+\sqrt{{k_{L\_LEAK}}^{2}+4\;k_{T\_LEAK}\;P_{PH}}}{2\;k_{T\_LEAK}}$$

From Equation (10), it is possible to obtain ${\dot{V}}_{LEAK}$ at any instant of time during spontaneous or mandatory breathing.

#### 2.4.3. ${\dot{V}}_{LEAK}$, ${\dot{V}}_{RES}$, and Tidal Volume (V_TID_) Calculation During Spontaneous Breathing

**Figure 6:**
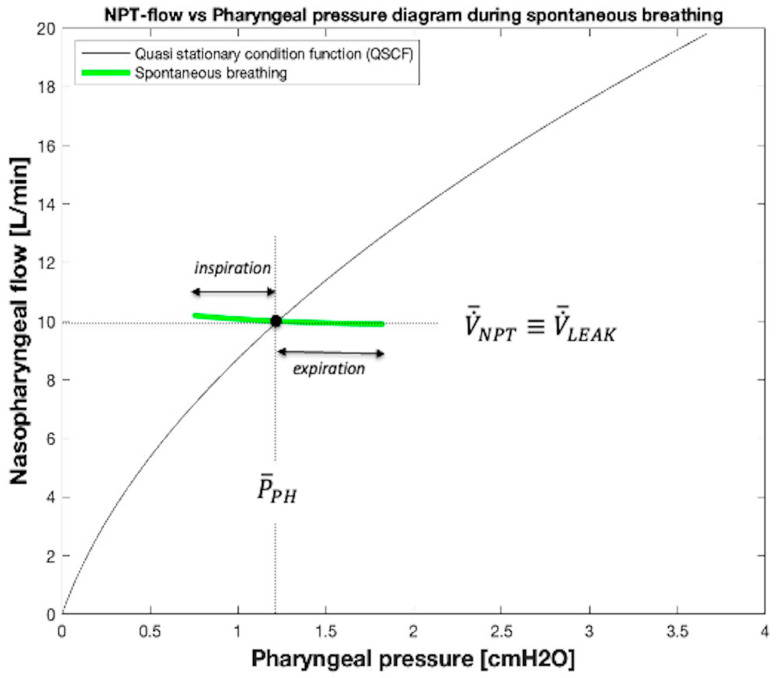
${\dot{V}}_{NPT}$ vs. *P_PH_* diagram during spontaneous breathing. The continuous line curve in this figure represents Equation (10) (Figure 5b, QSCF). The green segment is formed by the couple of pharyngeal pressure (*P_PH_*) and nasopharyngeal flow (${\dot{V}}_{NPT}$) values measured during spontaneous breathing.

Before a breath starts, there is a respiratory pause (i.e., apnea time), during which ${\dot{V}}_{NPT}$ and ${\dot{V}}_{LEAK}$ assume their equilibrium values (${\bar{\dot{V}}}_{NPT}$, ${\bar{\dot{V}}}_{LEAK}$), which coincide (${\bar{\dot{V}}}_{NPT}\;\equiv \;{\bar{\dot{V}}}_{LEAK}$). Because ${\bar{\dot{V}}}_{NPT}$ corresponds to the bias flow, we know exactly where the point is on the Cartesian diagram by crossing the bias flow line (${\bar{\dot{V}}}_{NPT}\;\equiv \;{\bar{\dot{V}}}_{LEAK}$) with the corresponding *P_PH_* line (${\bar{P}}_{PH}$, QSCF, Figure 6). When the patient starts breathing, the *P_PH_* decreases during inspiration and increases during expiration. The same pattern of increase and decrease should be seen with ${\dot{V}}_{NPT}$. However, the segment shown in Figure 6 appears almost horizontal, i.e., characterized by a very small amplitude of ${\dot{V}}_{NPT}$ oscillation around ${\bar{\dot{V}}}_{NPT}$ during each breath. This is because in most cases of NPT applications with FFODD in spontaneously breathing patients, *R_NPT_* is considerably greater than *R_LEAK_*. Consequently, with the patient’s respiratory system being like an ideal flow generator, the oscillation amplitude of ${\dot{V}}_{RES}$ occurring during the breathing is almost completely supplied by the oscillation amplitude of ${\dot{V}}_{LEAK}$. Furthermore, the features of this ideal flow generator do not depend on the capacitive (elastic) components of the patient’s respiratory system in such a way that all the pressures and flows appearing in Figure 4 (and therefore ${\dot{V}}_{NPT}$ and *P_PH_* also) are in phase with each other. This causes the typical hysteresis loop associated with an entire spontaneous breath to be reduced to a segment, also hiding the effect of non-linear *R_NPT_* and *R_LEAK_*.

To summarize, the oscillation amplitudes of the point that represents the current position in the ${\dot{V}}_{NPT}$ vs. *P_PH_* diagram during inspiration and expiration, i.e., the length of the inspiratory and expiratory portions of the segment shown in Figure 6, both depend mainly on the patient inspiratory effort and expiratory release, whereas the slope of the segment depends on the ratio between the values of *R_NPT_* and *R_LEAK_*, decreasing as the ratio decreases.

**Figure 7:**
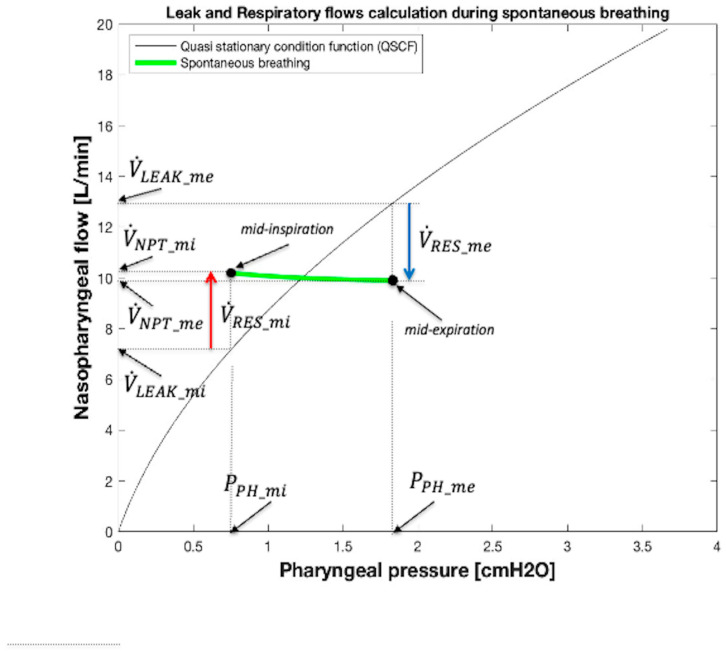
Graphical method to calculate ${\dot{V}}_{LEAK}$ and ${\dot{V}}_{RES}$ at mid-inspiration and mid-expiration times during spontaneous breathing. Starting from the point associated with mid-inspiration on the green segment, a vertical line is drawn to the x-axis until it intersects the QSCF (Equation (10), Figure 5b). From this point, a horizontal line is projected on the y-axis. The point of intersection represents ${\dot{V}}_{LEAK}$ at mid-inspiration. ${\dot{V}}_{RES}$ is calculated from Equation (1) as the difference between ${\dot{V}}_{NPT}$ and ${\dot{V}}_{LEAK}$ (red arrow). The blue arrow represents ${\dot{V}}_{RES}$ at mid-expiration.

From a graphic point of view (Figure 7), the current value of ${\dot{V}}_{LEAK}$ obtained from Equation (10) can be highlighted in the ${\dot{V}}_{NPT}$ vs. *P_PH_* diagram as the value of ${\dot{V}}_{NPT}$ corresponding to the intersection between the vertical projection passing through the current monitored value of *P_PH_* and the ${\dot{V}}_{LEAK}$ − *P_PH_* relationship during apnea (Equation (10), shown in Figure 5b, Figure 6, and Figure 7, QSCF). By way of example, Figure 7 shows the minimum (${\dot{V}}_{LEAK\_mi}$) and maximum (${\dot{V}}_{LEAK\_me}$) ${\dot{V}}_{LEAK}$ values calculated at mid-inspiration and mid-expiration times, respectively, when *P_PH_* assumes its minimum (*P_PH_mi_*) and maximum (*P_PH_me_*) values, respectively. Furthermore, in Figure 7 the values assumed by ${\dot{V}}_{NPT}$ at mid-inspiration (${\dot{V}}_{NPT\_mi}$) and mid-expiration (${\dot{V}}_{NPT\_mi}$) times, respectively, are also highlighted.

**Figure 8:**
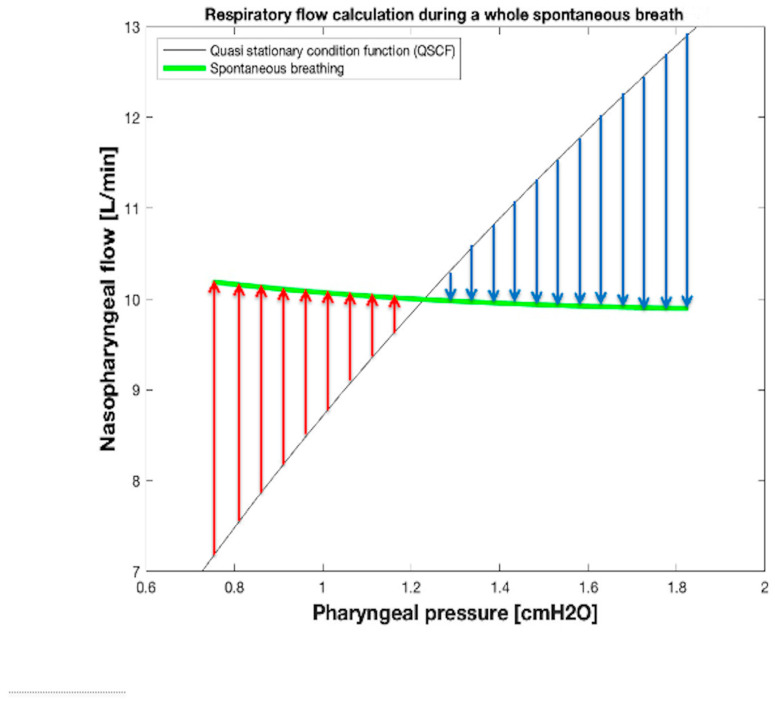
Graphical method to calculate ${\dot{V}}_{RES}$ at any time during spontaneous breathing. The length of each red or blue arrow indicates the inspiratory flow (${\dot{V}}_{INS}$) and expiratory flow (${\dot{V}}_{EXP}$) value at that point on the green segment.

Finally, during breathing, the current value of ${\dot{V}}_{RES}$ associated with the correspondent value of measured *P_PH_* can be computed considering Equation (1), i.e., as the difference between the current monitored value of ${\dot{V}}_{NPT}$ and the synchronized value of ${\dot{V}}_{LEAK}$ obtained by Equation (10). As an example, the lengths of the two vertical arrows ${\dot{V}}_{RES\_mi}$ and ${\dot{V}}_{RES\_me}$ reported in Figure 7 correspond to the maximum (${\dot{V}}_{RES\_mi}$, positive, red arrow) and minimum (${\dot{V}}_{RES\_me}$, negative, blu arrow) values of ${\dot{V}}_{RES}$ occuring at mid-inspiration and at mid-expiration times, respectively. The values of ${\dot{V}}_{RES}$ during a whole spontaneous breath, calculated in this way, are shown in Figure 8, where each amplitude of red and blue arrows equals the value assumed by inspiratory (${\dot{V}}_{INS}$) and expiratory (${\dot{V}}_{EXP}$) flows, respectively.

**Figure 9:**
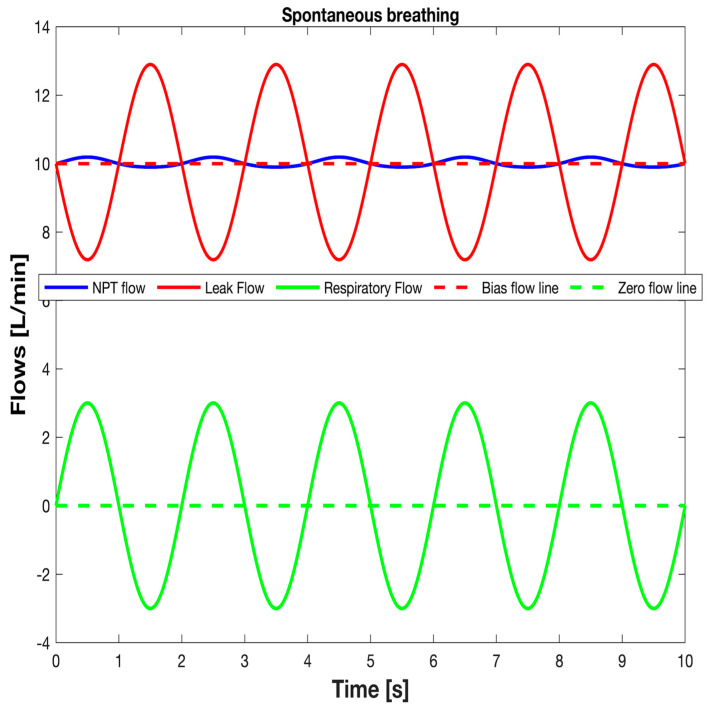
The typical waveforms over time of ${\dot{V}}_{NPT}$ (monitored), ${\dot{V}}_{LEAK}$ (calculated), and ${\dot{V}}_{RES}$ (calculated) during spontaneous breathing.

From what has been described so far in this section, it results that the ${\dot{V}}_{LEAK}$ and ${\dot{V}}_{RES}$ waveforms over time during spontaneous breathing can be computed and monitored real time by inserting into both Equations (1) and (10) the synchronized samples of the *P_PH_* and ${\dot{V}}_{NPT}$ waveforms over time available from monitoring system. Figure 9 shows the ${\dot{V}}_{NPT}$, ${\dot{V}}_{LEAK}$, and ${\dot{V}}_{RES}$ waveforms over time during spontaneous breathing obtained with the experimental setup reproducing the clinical scenario used for pediatric applications (Figure 7 and Figure 8). Finally, tidal volume (V_TID_) was calculated as ${\dot{V}}_{RES}$ integral over inspiratory time.

The assumption underlying the method described in the present section for ${\dot{V}}_{LEAK}$ and ${\dot{V}}_{RES}$ calculation is that the resistance of the leak channel (*R_LEAK_*) remains the same when switching from apnea to spontaneous breathing. This approach leads to correct results since, as already highlighted in Section 2.3., the breathing rate is so low (low speed of oscillation along the segment of Figure 6, Figure 7 and Figure 8) that the respiration process can be considered quasi stationary (QSC) according to the same condition for which the preliminary *P_PH_* − ${\dot{V}}_{LEAK}$ relationship during apnea was obtained.

#### 2.4.4. ${\dot{V}}_{LEAK}$, ${\dot{V}}_{RES}$, and V_TID_ Calculation During Mandatory Breathing

**Figure 10:**
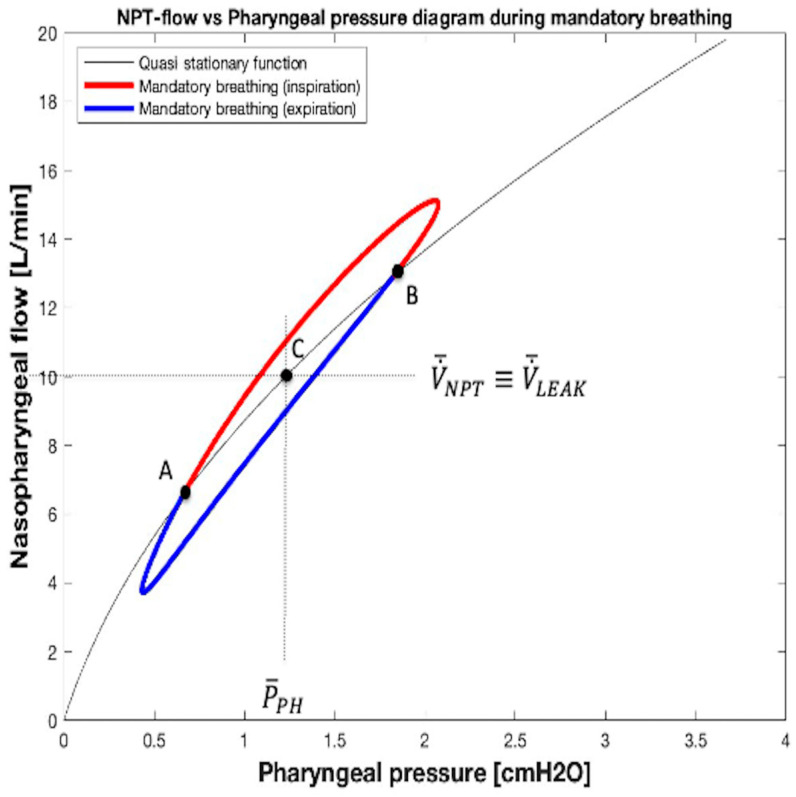
${\dot{V}}_{NPT}$ vs. *P_PH_* diagram during mandatory breathing. The continuous line curve in this figure represents Equation (10) (Figure 5b, quasi stationary function). Note that during mandatory breathing, the green segment in Figure 6 is now an ellipsoid. The three solid black dots A, B, and C mark the beginning of inspiration, beginning of expiration, and the equilibrium point set by bias flow (${\bar{\dot{V}}}_{NPT}\;\equiv \;{\bar{\dot{V}}}_{LEAK}$, ${\bar{P}}_{PH}$), respectively. At both points A and B, ${\dot{V}}_{RES}$ is zero.

When a patient remains apneic, manual mandatory breaths are supplied by the FFODD. In this case, a closed curve with ellipse-shaped (hysteresis loop) travelling clockwise appears in the ${\dot{V}}_{NPT}$ vs. *P_PH_* diagram. The geometrical center of the loop is the equilibrium point set by bias flow (${\bar{\dot{V}}}_{NPT}\;\equiv \;{\bar{\dot{V}}}_{LEAK}$) and corresponding to *P_PH_* value (${\bar{P}}_{PH}$) belonging to the ${\dot{V}}_{LEAK}\;$− *P_PH_* relationship at apnea (Figure 5b and Figure 10, quasi stationary function). An example of the loop related to a whole breath is shown in Figure 10 with two colors: red and blue.

The hysteresis loop is a representation of the mandatory breath and results, as such, due to the consequence of the capacitive (elastic) component, in the complex impedance that characterizes the (passive) patient’s respiratory system. Over time, the ${\dot{V}}_{NPT}$ waveform shifts compared to that of *P_PH_*. The length of the ellipse-shaped loop’s main axis is contingent on the amount of pumping provided with the balloon (FFPDD), while its slope is influenced by *R_LEAK_* and the patient’s respiratory system impedance. The length of the secondary axis of the ellipse-shaped loop, which quantifies the amount of hysteresis, is contingent on the ratio between the resistive and capacitive components that constitute the patient’s respiratory system impedance, as well as non-linear *R_NPT_* and *R_LEAK_*. During inspiration and expiration, the portion of the loop involved is the one marked in Figure 10 with the red and the blue colors, respectively. As in spontaneous breathing, the oscillation of *P_PH_* around ${\bar{P}}_{PH}$ occurring during mandatory breathing is normally associated with an oscillation of ${\dot{V}}_{NPT}$ around bias flow (${\bar{\dot{V}}}_{NPT}\;\equiv \;{\bar{\dot{V}}}_{LEAK}$) due to the changing of pressure drop across NPT.

**Figure 11:**
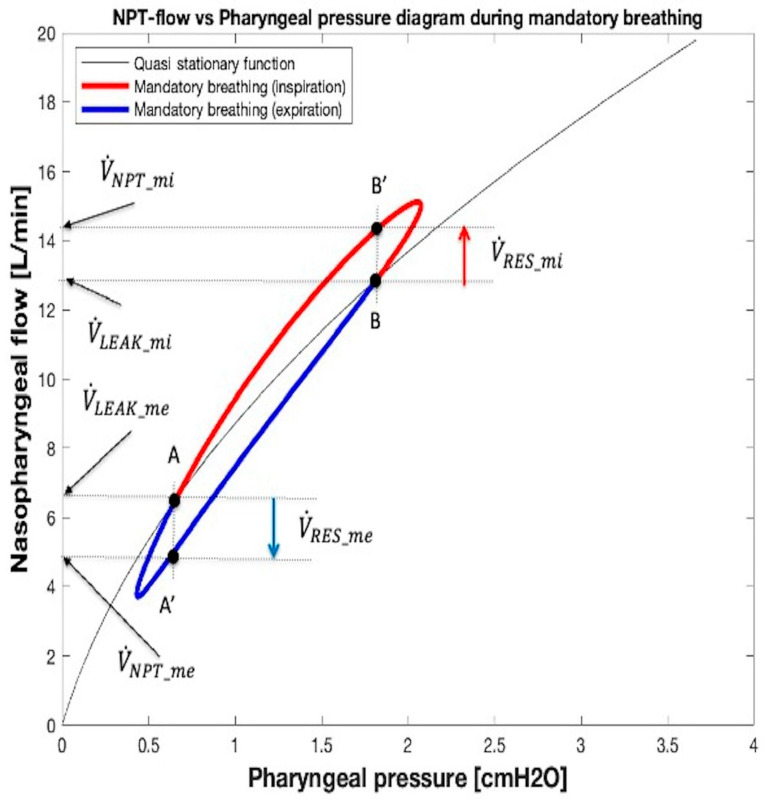
Graphical method to calculate ${\dot{V}}_{LEAK}$ and ${\dot{V}}_{RES}$ at mid-inspiration and mid-expiration times during mandatory breathing. Starting from point B’, associated with mid-inspiration on the red loop, a vertical line is drawn to the x-axis until it intersects the quasi stationary function (Equation (10), Figure 5b) at point B. From point B, a horizontal line is projected on the y-axis. The point of intersection represents ${\dot{V}}_{LEAK}$ at mid-inspiration. ${\dot{V}}_{RES}$ is calculated from Equation (1) as the difference between ${\dot{V}}_{NPT}$ and ${\dot{V}}_{LEAK}$ (red arrow). The blue arrow represents ${\dot{V}}_{RES}$ at mid-expiration (point A’ and its projection on the loop, point A).

As in spontaneous breathing, from a graphic point of view (Figure 11), the current value of ${\dot{V}}_{LEAK}$ obtained from Equation (10) can be highlighted in the ${\dot{V}}_{NPT}$ vs. *P_PH_* diagram as the value of ${\dot{V}}_{NPT}$ corresponding to the intersection between the vertical projection passing through the current monitored value of *P_PH_* and the ${\dot{V}}_{LEAK}$ − *P_PH_* relationship during apnea (Equation (10) shown in Figure 5b, Figure 10 and Figure 11, quasi stationary function). By way of example, Figure 11 shows the minimum (${\dot{V}}_{LEAK\_mi}$) and maximum (${\dot{V}}_{LEAK\_me}$) ${\dot{V}}_{LEAK}$ values calculated at mid-inspiration and mid-expiration times, respectively. Furthermore, in Figure 11, the values assumed by ${\dot{V}}_{NPT}$ at mid-inspiration (${\dot{V}}_{NPT\_mi}$) and mid-expiration (${\dot{V}}_{NPT\_mi}$) times, respectively, are also highlighted.

**Figure 12:**
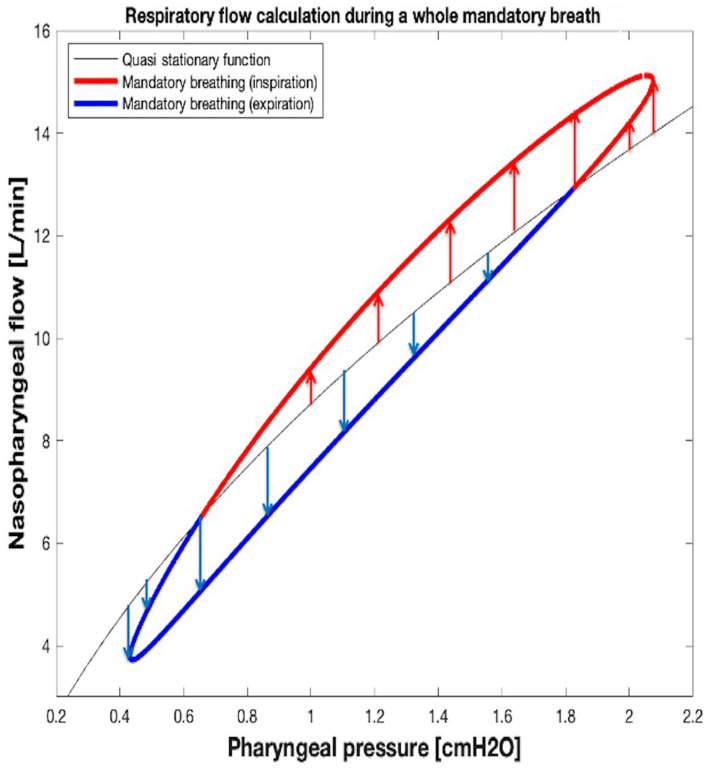
Graphical method to calculate ${\dot{V}}_{RES}$ at any time during mandatory breathing. The length of each red or blue arrow indicates the inspiratory flow (${\dot{V}}_{INS}$) and expiratory flow (${\dot{V}}_{EXP}$) value at that point on the loop.

Finally, during breathing, the current value of ${\dot{V}}_{RES}$ associated with the correspondent value of monitored *P_PH_* can be computed considering Equation (1), i.e., as the difference between the current monitored value of ${\dot{V}}_{NPT}$ and the synchronized value of ${\dot{V}}_{LEAK}$ obtained by Equation (10). As an example, the length of the two vertical arrows ${\dot{V}}_{RES\_mi}$ and ${\dot{V}}_{RES\_me}$ reported in Figure 11 correspond to the maximum (positive, red arrow) and minimum (negative, blue arrow) values of ${\dot{V}}_{RES}$ occuring at mid-inspiration and at mid-expiration times, respectively. The values of ${\dot{V}}_{RES}$ during a whole mandatory breath, calculated in this way, are shown in Figure 12, where each amplitude of red and blue arrows equals the value assumed by inspiratory (${\dot{V}}_{INS}$) and expiratory (${\dot{V}}_{EXP}$) flows, respectively.

**Figure 13:**
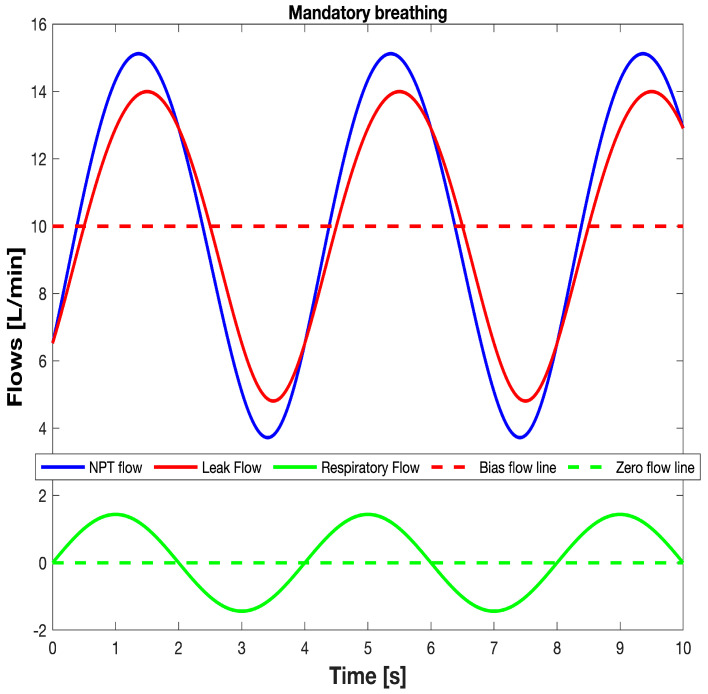
The typical waveforms over time of ${\dot{V}}_{NPT}$ (monitored), ${\dot{V}}_{LEAK}$ (monitored), and ${\dot{V}}_{RES}$ (calculated) during mandatory breathing.

From what has been described so far in this section, it results that the ${\dot{V}}_{LEAK}$ and ${\dot{V}}_{RES}$ waveforms over time during mandatory breathing can be computed and monitored real time by inserting into both the Equations (10) and (1) the synchronized samples of the *P_PH_* and ${\dot{V}}_{NPT}$ waveforms over time available from monitoring system. Figure 13 shows the ${\dot{V}}_{NPT}$, ${\dot{V}}_{LEAK}$, and ${\dot{V}}_{RES}$ waveforms over time during mandatory breathing (Figure 11 and Figure 12). Finally, tidal volume (V_TID_) was calculated as the ${\dot{V}}_{RES}$ integral over inspiratory time.

Again, we assumed that the resistance of the leak channel (*R_LEAK_*) remains the same when switching from apnea to mandatory breathing. The approach described in the present section leads to correct results, since, as already highlighted in Section 2.3., the breathing rate is so low (low travel speed along the loop of Figure 10, Figure 11 and Figure 12) that the respiration process can be considered quasi stationary according to the same condition (QSC) for which the preliminary *P_PH_* − ${\dot{V}}_{LEAK}$ relationship during apnea was obtained.

### 2.5. Statistical Analysis

The hypothesis we formulated at the beginning of the test was that the measured V_TID_ (V_TID_M_) and the calculated V_TID_ (V_TID_C_) data belong to the same population (null hypothesis). In order to verify the validity of the null hypothesis, we subjected the data obtained for both the variables V_TID_M_ and V_TID_C_ to the paired t Student two-tailed test which is based on the combined estimate of the variance. The critical threshold values of t (t*) associated with the significance level (α) were equal to 0.01 and with the appropriate value of the degrees of freedom (ν) equal to 70 (corresponding to 36 samples for both observed variables: t*0.01 = 2.648). As is known, if the absolute value of t calculated from the data observed is greater than t*0.01, the null hypothesis is to be considered incorrect by accepting that the probability of committing an error is less than or equal to 1%. The test was carried out for all combinations of nominal tidal volume and leak size covering the entire range of clinical interest for NPV applications.

## 3. Results

In this study, we developed a method to calculate ${\dot{V}}_{LEAK}$, ${\dot{V}}_{RES}$, and V_TID_ during NPV. Given that NPV operates as an open system, the flow delivered to the patient (${\dot{V}}_{NPT}$) must be adjusted by subtracting the flow lost through the mouth (leakage channel, ${\dot{V}}_{LEAK}$) to accurately determine the ${\dot{V}}_{RES}$. The procedure for calculating ${\dot{V}}_{LEAK}$, and thus ${\dot{V}}_{RES}$, consisted of several steps. First, the relationship between *P_PH_* and ${\dot{V}}_{LEAK}$ under apneic conditions was characterized. It is noteworthy that this relationship can only be characterized during apnea when the ${\dot{V}}_{LEAK}$, i.e., the unknown variable, is equal to the flow delivered through the NPT (${\dot{V}}_{NPT})$. The relationship was then interpolated by Rohrer’s equation (Equation (8)) to obtain a curve defined by the coefficients *k_L_LEAK_* and *k_T_LEAK_* (Figure 5a). Subsequent to this preliminary yet crucial step, the ${\dot{V}}_{LEAK}$ was determined using Equation (10), with the coefficients *k_L_LEAK_* and *k_T_LEAK_* being those calculated in the initial step. According to Equation (1), the ${\dot{V}}_{RES}$ was obtained from the difference between ${\dot{V}}_{NPT}$ and ${\dot{V}}_{LEAK}$, and the V_TID_ as the integration of the ${\dot{V}}_{RES}$ over the inspiratory time. All the measurement data we obtained were analyzed by the Shapiro–Wilk test, which confirmed that they belong to a normal distribution. Consequently, for any statistical analysis, we considered the following parameters relative to our target variable, i.e., the lung volume inspired by the patient during each breath, (i.e., tidal volume (V_TID_)): mean (M), standard deviation (SD), and standard error on the mean (SEM).

The accuracy and precision errors associated with the IngMar ASL 5000 Breathing Simulator were assessed as relative error (RE) and coefficient of variation (CV) of V_TID_. The ER of V_TID_ was calculated in a leak-free system with no background flow and defined by RE (%) = 100 × (M − nominal V_TID_)/nominal V_TID_. Nominal V_TID_ (V_TID_N_) is the value of V_TID_ set for delivering each predetermined value. A percentage of less than 3% was considered acceptable [15]. The CV of V_TID_, defined by CV (%) = 100 × (SD/M), was used to describe the reproducibility for V_TID_. The resulting REs and CVs of V_TID_ were 0.4% and 0.8%, respectively.

The accuracy and precision errors associated with the experimental setup and the acquisition/elaboration software (PicoScope/MATLAB, version R2024b) are reported elsewhere [18].

### 3.1. Results of Coefficient Determination (k_L_LEAK_ and k_T_LEAK_)

We considered three leak channels with different amounts of flow loss (small, medium, and large leak size) for pediatric and adult applications. The coefficients obtained using the ${\dot{V}}_{LEAK}$ − *P_PH_* relationship during apnea, which was analytically interpolated with a second-order polynomial (Equation (8)), are reported in Table 1.

### 3.2. Tidal Volume Computing and Validation

To evaluate the accuracy of the algorithm for determining ${\dot{V}}_{LEAK}$, ${\dot{V}}_{RES}$, and V_TID_ during NPV, we compared the V_TID_M_ measured from ${\dot{V}}_{RES}$ detected by the PNT_C (Figure 3) with the V_TID_C_ calculated using the algorithm.

For each selected V_TID_N_, 36 small leak (SL), 36 medium leak (ML) and 36 large leak (LL) measurements were performed. The data obtained at the end of each measurement consist of the average value of both V_TID_M_ and V_TID_C_ obtained by collecting 1000 consecutive breaths. Normal distribution was tested using the Shapiro–Wilk test. Since the data were normally distributed, for statistical analysis we considered the mean and the standard deviation of both variables studied (V_TID_C_ and V_TID_M_). Assuming that the type of method used is the only factor affecting the data, the statistical assessment of each comparison at different V_TID_N_ and leak size between V_TID_C_ and V_TID_M_ was performed using the one-way analysis of variance (ANOVA) in the version derived for two groups or variables (paired Student *t* test).

#### 3.2.1. Validation of Tidal Volume Computation Using the Algorithm for of Spontaneous Breathing

To simulate pediatric applications, with a bias flow and R_V_ setting of 16 L/min and 60 cmH_2_O/L/s in all cases, we independently varied the amplitude of ${\dot{V}}_{INS}$ (A) from 1 to 20 L/min and the duration of the inspiration (TI) from 0.5 to 1 s in order to obtain three target values of V_TID_N_: 10, 50, and 100 mL delivered through the 3.0, 4.0, and 5.0 mm ID, respectively.

To simulate adult applications, with a bias flow and R_V_ setting of 32 L/min and 60 cmH_2_O/L/s in all cases, we independently varied A from 10 to 40 L/min and TI from 1 to 2 s in order to obtain three target values of V_TID_N_: 200, 300, and 400 mL delivered through the 7.0, 8.0, and 9.0 mm ID, respectively.

The results of the statistical analysis are reported in Table 2 and Table 3.

#### 3.2.2. Validation of Tidal Volume Computation Using the Algorithm for Mandatory Ventilation

In this case, as already mentioned in Section 2.4.4., with the patient’s respiratory system completely passive, the resulting V_TID_ depends on the amplitude of the pumped flow (PF) generated by the anesthetist through their action on the inflated balloon and its time duration (TI), but also on ID size, leak size, patient’s total inspiratory resistance (R_P_), and compliance (C_P_). The bias flow and R_V_ should be set to ensure adequate inflating of the balloon in the required time to the desired V_TID_.

To simulate pediatric applications, with a bias flow and R_V_ setting of 16 L/min and 60 cmH_2_O/L/s in all cases, we independently varied PF from 1 to 20 L/min and TI from 0.5 to 1 s in order to obtain three target values of V_TID_N_: 10, 50, and 100 mL delivered through the 3.0, 4.0, and 5.0 mm ID, to a patient with R_P_ = 100 cmH_2_O/L/s and C_P_ = 10 mL/cmH_2_O, with R_P_ = 50 cmH_2_O/L/s and C_P_ = 20 mL/cmH_2_O and with R_P_ = 20 cmH_2_O/L/s and C_P_ = 50 mL/cmH_2_O, respectively.

To simulate adult applications properly, with a bias flow and R_V_ setting of 32 L/min and 60 cmH_2_O/L/s in all cases, we independently varied PF from 1 to 40 L/min and TI from 1 to 2 s in order to obtain three target values of V_TID_N_: 200, 300, and 400 mL delivered through the 7.0, 8.0, and 9.0 mm ID, respectively. For all patients, R_P_ and C_P_ were set to 10 cmH_2_O/L/s and 100 mL/cmH_2_O, respectively.

The results of the statistical analysis are reported in Table 4 and Table 5.

## 4. Discussion

In response to the clinical demand for a new procedure capable of assessing the real value of the tidal volume inspired by patients while they are assisted with NPV, a new computational method was developed. The purpose of this method is to calculate tidal volume in conditions where direct measurement is otherwise difficult to achieve. Direct measurement of tidal volume necessitates the implementation of a pneumotachograph at the extremity of the leak channel. Given the prevalence of NPV-demanding procedures, such as gastroscopies in small children, the employment of an orofacial mask equipped with a pneumotachograph is impractical, as it would interfere with the actions of physicians.

Any method that aims to monitor the effective value of tidal volume in an open system of ventilation must first determine the ${\dot{V}}_{LEAK}$ waveform during breathing. This objective can be realized if airflow resistance of the leak channel can be accurately evaluated over the entire range of flow values that occur during breathing. The method proposed in this paper includes two steps. The initial step in the procedure is to characterize the relationship between the *P_PH_* and ${\dot{V}}_{LEAK}$, which allows calculation of the coefficients of the interpolated curve according to Rohrer’s equation. As ${\dot{V}}_{LEAK}$ cannot always be measured directly in the patient without a PNT and a mask, it is nevertheless possible to ascertain the relationship between *P_PH_* and ${\dot{V}}_{LEAK}$ because there is a period (when the patient is not breathing, i.e., apnea) when ${\bar{\dot{V}}}_{NPT}\;\equiv \;{\bar{\dot{V}}}_{LEAK}$. In the second step of the method, an equation is devised that links the ${\dot{V}}_{LEAK}$, the ${\dot{V}}_{NPT}$, the *P_PH_*, and the coefficients calculated in the first step during the apnea. This equation is derived during breathing that is either spontaneous or mandatory. After obtaining the ${\dot{V}}_{LEAK}$ waveform, the ${\dot{V}}_{RES}$ waveform is calculated as the difference between ${\dot{V}}_{NPT}$ and ${\dot{V}}_{LEAK}$. At this point, V_TID_ is obtained from the integration during the inspiratory time of the difference between ${\dot{V}}_{NPT}$ and ${\dot{V}}_{LEAK}$.

To the best of our knowledge, our computational method is the first to be developed and tested for tidal volume determination during NPV.

Testing the method’s efficacy involved a comparison between the tidal volume obtained by measurement with a pneumotachograph positioned at tracheal level in the lung simulator (V_TID_M_), universally regarded as the gold standard, and the tidal volume computed with the algorithm (V_TID_C_).

The criteria chosen to properly evaluate the reliability of the results obtained with the algorithm (V_TID_C_) compared to those obtained with the gold standard (V_TID_M_) for different values of nominal tidal volume (V_TID_N_) and leak size typical for pediatric and adult applications, both for spontaneous and mandatory breaths, showed a similar magnitude of error. Specifically, the reported data show that in no case does the absolute value of t exceed t*0.01, indicating that there is no statistically significant difference between V_TID_C_ and V_TID_M_ in any of the cases studied. From this result, it follows that the null hypothesis can be accepted, confirming the validity of the algorithm presented here compared to the gold standard. In other words, the reliability of the results obtained with the algorithm is comparable to that obtained with the gold standard (i.e., with a pneumotachograph placed downstream of the leak).

### 4.1. Other Methods to Measure Tidal Volume During Nasal Ventilation

In the neonatal population, intermittent positive pressure ventilation administered via nasal mask or prongs, short or long, single or bi-nasal, is a prevalent clinical practice. The necessity of monitoring tidal volume in infants with acute respiratory failure or in those with healthy lungs during procedural sedation arises due to its correlation with clinical outcomes. The quantification of tidal volume in patients under NPV poses a significant challenge. Numerous clinical studies have examined direct or indirect methods, but the results have been disappointing. For example, Matlock et al. measured tidal volume transmission during non-synchronized nasal intermittent positive pressure ventilation by respiratory inductance plethysmography in a group of preterm infants [20]. With this method, mean tidal volumes were expressed in arbitrary units, which makes comparisons with other studies impossible. In a recent study in adult patients, Janssen and colleagues endeavored to ascertain the validity of ultrasound measurements of diaphragm excursion as a surrogate marker for tidal volume in patients with respiratory failure. However, a notable disparity emerged between the observed and predicted tidal volumes. Furthermore, substantial variability was observed in tidal volume estimation among study participants. Consequently, the reliability of quantifying absolute tidal volume from diaphragm excursion remains questionable [21]. Lenhardt et al. tested the hypothesis that nasopharyngeal ventilation is superior in its efficacy compared to traditional facemask ventilation. The primary outcome was tidal volume. In this case, tidal volume was measured with a pneumotachograph via a method aiming to minimize the ${\dot{V}}_{LEAK}$ (simultaneous sealing of the mouth and nares) which could be unfeasible in other contexts [3]. More sophisticated studies come from the group of Schmalisch and colleagues, who compared ${\dot{V}}_{LEAK}$ data obtained using different leak modeling and in vitro measurements [12]. Fischer et al. investigated tidal volume and leak measurements during CPAP in neonates using a commercial ventilatory device equipped with a flow sensor at the Y-piece. During nasopharyngeal CPAP, leak and tidal volumes were measured using the commercial ventilator and an algorithm to correct measured tidal volume in the presence of leaks. Leak and corrected tidal volume could be determined in the presence of leaks of up to 69%, but leaks during CPAP often exceeded the measuring range. The authors therefore concluded that advanced equipment was necessary to further investigate the effects of leaks on neonatal CPAP therapy [9].

### 4.2. Clinical Implications

The experimental results of the present study provide a basis for conducting clinical trials to determine the validity of the proposed method in patient populations supported by NPV. In the context of upper gastrointestinal endoscopy, the implementation of the mask with the pneumotachograph has the potential to interfere with the movements of the endoscopist. This in turn adds another layer of complexity to the procedure.

### 4.3. Limitations

The method is based on the premise that the airflow resistance of the leak channel during both spontaneous and mandatory breathing is nearly the same as that calculated in the apnea condition. It also assumes that the patient’s respiratory mechanics are linear during mandatory ventilation. The validity of these assumptions may not always be guaranteed in vivo. In this study, a healthy lung model was utilized, as the primary application of NPV is in procedural sedation. Further studies are currently being conducted to investigate potential differences in cases involving diseased lungs.

## 5. Conclusions

The findings documented in this study demonstrate the validity and potential usefulness of the algorithm devised for the real-time detection of leak flow (${\dot{V}}_{LEAK}$), respiratory flow (${\dot{V}}_{RES}$), and tidal volume (V_TID_) waveforms during NPV. This algorithm has the potential to provide anesthetists with a valuable instrument to optimize the control of both zaspontaneous and mandatory breathing during nasopharyngeal ventilation through accurate tidal volume estimation in patients. Further studies will be conducted to verify the in vivo feasibility of this method.

## Figures and Tables

**Table 1:** Results of *k_L_LEAK_* and *k_T_LEAK_* at different leak sizes. The measure of how closely the *P_PH_* − ${\dot{V}}_{LEAK}$ relationship matches the fitted equation (Equation (8)) is expressed as GOF.

Leak Size	*k_L_LEAK_* [cmH_2_O s/L]	*k_T_LEAK_* [cmH_2_O s^2^/L^2^]	GOF MSE; NRMSE; NMSE
Small (SL)	11.88	66.88	0.002; 0.988; 0.999
Medium (ML)	6.12	32.71	0.002; 0.981; 0.999
Large (LL)	3.66	17.34	0.003; 0.976; 0.999

GOF: goodness of fit; MSE: mean squared error [cmH_2_O]; NRMSE: normalized root mean squared error [adimensional]; NMSE: normalized mean squared error [adimensional].

**Table 2:** Statistical data related to tidal volume (V_TID_) computation for spontaneous breathing.

V_TID_N_/NPT ∅		V_TID_M_/V_TID_C_ M (SD)	
Pediatric	Small Leak (SL)	Medium Leak (ML)	Large Leak (LL)
10/3.0 mm	9.92 (0.16)/9.90 (0.26)	9.95 (0.11)/9.89 (0.26)	9.94 (0.28)/9.89 (0.26)
50/4.0 mm	49.86 (1.33)/49.24 (1.31)	49.85 (1.32)/49.22 (1.31)	49.78 (1.33)/99.90 (1.31)
100/5.0 mm	99.87 (1.66)/98.43 (2.62)	99.69 (1.86)/98.38 (2.62)	99.34 (0.98)/98.33 (2.62)
**Adult**			
200/7.0 mm	198.44 (2.88)/198.15 (5.28)	198.87 (3.28)/198.05 (7.27)	198.44 (3.88)/197.80 (5.27)
300/8.0 mm	298.62 (5.08)/302.14 (8.15)	289.62 (6.08)/300.98 (8.56)	301.62 (5.08)/305.60 (8.14)
400/9.0 mm	398.78 (8.94)/405.21 (10.78)	410.78 (10.94)/405.01 (10.78)	410.78 (10.94)/404.50 (10.77)

V_TID_N_: nominal V_TID_; V_TID_M_: measured V_TID_; V_TID_C_: calculated V_TID_; All data are in milliliters [mL]; M: mean; SD: standard deviation.

**Table 3:** Results of statistical comparison at different values of V_TID_N_ between V_TID_M_ and V_TID_C_ for spontaneous breathing simulation (paired Student *t* test).

V_TID_N_/NPT ∅		t	
Pediatric	Small Leak (SL)	Medium Leak (ML)	Large Leak (LL)
10/3.0 mm	0.3989	0.4788	0.5587
50/4.0 mm	1.9925	2.0724	2.1523
100/5.0 mm	2.3102	2.3901	2.4700
**Adult**			
200/7.0 mm	0.2392	0.3190	0.5188
300/8.0 mm	−1.3155	−1.2357	−1.0360
400/9.0 mm	2.1755	2.2553	2.4550

V_TID_N_: nominal V_TID_ in milliliters [mL].

**Table 4:** Statistical data related to tidal volume (V_TID_) computation for mandatory breathing.

V_TID_N_/NPT ∅		V_TID_M_/V_TID_C_ M (SD)	
Pediatric	Small Leak (SL)	Medium Leak (ML)	Large Leak (LL)
10/3.0 mm	10.3 (0.62)/9.89 (0.26)	9.95 (0.51)/9.12 (0.37)	9.89 (0.19)/9.34 (1.26)
50/4.0 mm	49.01 (2.31)/49.12 (1.31)	50.13 (2.32)/49.22 (1.31)	51.68 (5.13)/49.90 (2.38)
100/5.0 mm	102.45 (2.88)/97.35 (2.03)	103.89 (4.86)/98.38 (3.25)	100.44 (4.99)/98.56 (2.92)
**Adult**			
200/7.0 mm	199.87 (3.78)/195.89 (5.28)	200.87 (6.18)/198.10 (6.23)	194.14 (3.67)/197.80 (5.27)
300/8.0 mm	300.90 (7.48)/304.14 (6.67)	289.12 (6.89)/303.98 (8.90)	305. 23 (4.16)/305.60 (8.14)
400/9.0 mm	397.10 (8.94)/406.23 (9.88)	406.12 (5.84)/407.10 (10.88)	410.18 (9.64)/404.50 (10.77)

V_TID_N_: nominal V_TID_; V_TID_M_: measured V_TID_; V_TID_C_: calculated V_TID_; All data are in milliliters [mL]; M: mean; SD: standard deviation.

**Table 5:** Results of statistical comparison at different values of V_TID_N_ between V_TID_M_ and V_TID_C_ for mandatory breathing simulation (paired Student *t* test).

V_TID_N_/NPT ∅		t	
Pediatric	Small Leak (SL)	Medium Leak (ML)	Large Leak (LL)
10/3.0 mm	−1.2698	−1.2698	−1.4278
50/4.0 mm	−2.0529	−2.0529	−2.2109
100/5.0 mm	1.7266	2.5058	2.3478
**Adult**			
200/7.0 mm	−1.0325	−1.1116	−1.2303
300/8.0 mm	−1.8005	−1.8796	−1.9982
400/9.0 mm	2.3484	2.2693	2.1506

V_TID_N_: nominal V_TID_ in milliliters [mL].

## Data Availability

Data are contained within the article. Further data that support the findings of this study are available upon request from the corresponding author.
